# SQOR as a metabolic rheostat of H_2_S: structure, redox homeostasis, and disease therapy

**DOI:** 10.3389/fcell.2025.1685252

**Published:** 2025-10-23

**Authors:** Ming-Hui Peng, Kai-Lun Zhang, Zhong-Wu Ma, He-Wei Zhang, Shi-Wei Guan, Hai-Bo Yu

**Affiliations:** Department of Hepatobiliary Surgery, The Wenzhou Central Affiliated Hospital of Wenzhou Medical University, Wenzhou, Zhejiang, China

**Keywords:** sulfide:quinone oxidoreductase (SQOR), hydrogen sulfide (H_2_S), ferroptosis, coenzyme Q10, colorectal cancer, ulcerative colitis, sulfide oxidation pathway, mitochondrial bioenergetics

## Abstract

Sulfide:quinone oxidoreductase (SQOR) is an inner-mitochondrial-membrane enzyme that couples hydrogen sulfide oxidation to the coenzyme Q pool, thereby linking sulfur metabolism with cellular bioenergetics and redox control. Recent structural and mechanistic advances—most notably the catalytic cysteine trisulfide—clarify how membrane context and substrate availability tune catalytic flux, yet debate persists over the physiological sulfur acceptor (glutathione versus sulfite) and how microenvironments route sulfide. SQOR also shapes ferroptosis: by using hydrogen selenide to reduce ubiquinone, it elevates ubiquinol and suppresses lipid peroxidation independently of glutathione peroxidase-4. We synthesize cross-system disease evidence—brain (hypoxia/ischemia, neuroinflammation), heart (divergent roles in acute ischemia–reperfusion versus chronic failure), kidney (mitochondrial dysfunction and cGAS–STING(cyclic GMP–AMP synthase–stimulator of interferon genes)–driven fibrosis), gastrointestinal tract (stage-specific effects in colorectal cancer and impaired detoxification in ulcerative colitis), bone/metabolic disorders, and the male reproductive system—highlighting SQOR’s bidirectional pathology when hydrogen sulfide is excessive or depleted. Viewing SQOR as a “metabolic rheostat” reconciles these paradoxes and underscores therapeutic opportunities: metabolic supplementation (e.g., coenzyme Q10), selective inhibition or activation, and context-matched modulation. We further propose companion diagnostics that quantify sulfur/selenium species and enzyme activity to enable patient stratification and de-risk clinical translation.

## Introduction

Hydrogen sulfide (H_2_S) is a toxic gas that was traditionally viewed as an environmental pollutant (Mustafa et al., 2009), but recent studies have found it plays an important role in physiological signaling (Wang, 2002; Kimura et al., 2012). As the third gaseous signaling molecule after nitric oxide and carbon monoxide (Abe and Kimura, 1996; Cirino et al., 2023), H_2_S is produced endogenously in mammals by enzymes such as cystathionine β-synthase (CBS), cystathionine γ-lyase (CSE), and 3-mercaptopyruvate sulfurtransferase (3-MST; also called MPST). It is widely involved in regulating vasodilation (Sekiguchi et al., 2014; Hosoki et al., 1997; Zhao and Wang, 2002), neurotransmission (Tang et al., 2008; Fan et al., 2013; Xuan et al., 2012), cell proliferation (Wang R-H. et al., 2024; Panza et al., 2022), and antioxidant defenses (Gupta et al., 2022; Jiang et al., 2025), among other biological processes. However, the actions of H_2_S are strictly concentration-dependent, acting as a double-edged sword: at physiological levels it functions as a beneficial signaling molecule, whereas at high concentrations it becomes strongly cytotoxic by inhibiting cytochrome c oxidase (Complex IV) at the end of the mitochondrial respiratory chain, leading to energy depletion (Caro et al., 2011; Mallardi et al., 2025). This dual nature makes the role of H_2_S in health and disease extremely complex, requiring the body to maintain H_2_S within a narrow steady-state window via a sophisticated metabolic regulatory system (Yang, 2011; Zhao et al., 2023).

**FIGURE 1:**
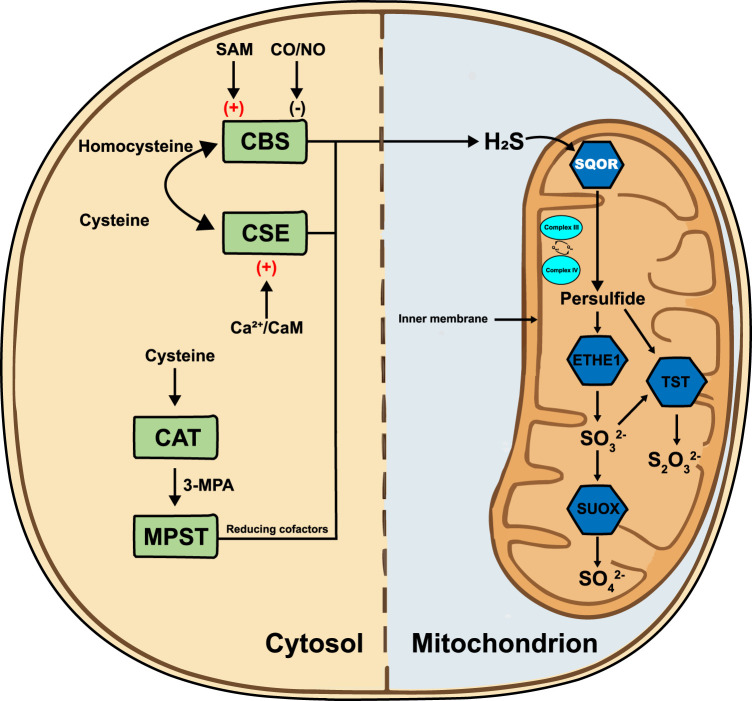
H_2_S metabolic pathway. In the cytosol, cystathionine β-synthase (CBS) and cystathionine γ-lyase (CSE) convert homocysteine and cysteine to H_2_S; CBS is stimulated by S-adenosylmethionine (SAM) and inhibited by carbon monoxide/nitric monoxide (CO/NO), whereas CSE is activated by Ca^2+^/calmodulin (Ca^2+^/CaM). Cysteine aminotransferase (CAT) and 3-mercaptopyruvate sulfurtransferase (MPST) generate H_2_S via 3-mercaptopropionic acid (3-MPA) with reducing cofactors. H_2_S diffuses into the mitochondrion, where sulfide:quinone oxidoreductase (SQOR) is anchored to the inner mitochondrial membrane (IMM). SQOR oxidizes H_2_S and transfers electrons to the ubiquinone pool (Q_red_/Q_ox_) adjacent to Complex III and Complex IV of the respiratory chain. The resulting persulfide is metabolized by persulfide dioxygenase (ETHE1) to sulfite (SO_3_^2-^), by thiosulfate sulfurtransferase (TST) to thiosulfate (S_2_O_3_^2-^) and ultimately by sulfite oxidase (SUOX) to sulfate (SO_4_^2-^). H_2_S, hydrogen sulfide; SAM, S-adenosylmethionine; CO/NO, carbon monoxide/nitric monoxide; CBS, cystathionine β-synthase; CSE, cystathionine γ-lyase; Ca^2+^/CaM, calcium/calmodulin complex; CAT, cysteine aminotransferase; 3-MPA, 3-mercaptopropionic acid; MPST, 3-mercaptopyruvate sulfurtransferase; SQOR, sulfide:quinone oxidoreductase; Q_red_/Q_ox_, oxidized/reduced ubiquinone; Complex III, ubiquinol-cytochrome c oxidoreductase; Complex IV, cytochrome c oxidase; ETHE1, persulfide dioxygenase; TST, thiosulfate sulfurtransferase; SUOX, sulfite oxidase; SO_4_^2-^, sulfate; SO_3_^2-^, sulfite; S_2_O_3_^2-^, thiosulfate.

In the H_2_S metabolic regulatory network, sulfide:quinone oxidoreductase (SQOR) is a crucial rate-limiting enzyme (Kanemaru et al., 2024; Kumar et al., 2024). SQOR is anchored to the inner mitochondrial membrane and catalyzes the oxidation of H_2_S to chemically more stable persulfides, the first and irreversible step of the sulfide oxidation pathway. In this process, SQOR directly transfers electrons from H_2_S oxidation to the coenzyme Q (CoQ) pool in the electron transport chain, tightly coupling H_2_S removal with cellular energy production and redox homeostasis (Landry et al., 2017). Early literature therefore portrayed SQOR primarily as a detoxifying enzyme that prevents H_2_S-mediated inhibition of complex IV, as illustrated by the severe metabolic crises seen in SQOR deficiency (Kanemaru et al., 2024; Friederich et al., 2020). Recent evidence, however, highlights that beyond detoxification, SQOR integrates sulfide oxidation with ubiquinone redox cycling, modulates mitochondrial respiratory capacity, and generates hydropersulfides and thiosulfate that act as radical-trapping antioxidants (Lee et al., 2025). This shift in perspective frames SQOR as a metabolic rheostat that dynamically tunes sulfur flux and redox balance according to cellular needs. Through this unique mechanism, SQOR not only efficiently removes potentially toxic H_2_S (preventing its accumulation in mitochondria), but also plays a central regulatory role in maintaining cellular energy balance and managing oxidative stress, serving as a key junction between sulfur metabolism and bioenergetics (Salti et al., 2024). Figure 1 illustrates the H_2_S metabolic pathway.

Given these core functions, it is not surprising that SQOR dysfunction is closely associated with the onset and progression of various human diseases (Youness et al., 2021; Youness et al., 2024; Kimura, 2014; Stein and Bailey, 2013), and its effects often exhibit a high degree of situational dependence. In cancer, SQOR expression levels vary across different stages and tumor types, influencing tumor growth and survival by regulating H_2_S metabolism and cellular sensitivity to ferroptosis (Youness et al., 2018; Lee et al., 2024). In cardiovascular diseases, SQOR has dual functionality: in acute injuries such as myocardial ischemia–reperfusion, SQOR activation is protective, whereas in chronic heart failure, SQOR inhibition may be beneficial by increasing endogenous H_2_S levels (Nishimura et al., 2025; Luo et al., 2021; Salloum et al., 2015). In neurodegenerative diseases, SQOR dysfunction is linked to toxic H_2_S accumulation and neuroinflammation, and SQOR gene mutations can directly cause fatal Leigh syndrome (Gheibi et al., 2014; Yang et al., 2022; Ji et al., 2016). In metabolic disorders such as diabetic nephropathy, changes in SQOR expression and activity are also closely tied to disease progression (Kanemaru et al., 2024; Lee et al., 2025; Bushell et al., 2023). Under the metabolic rheostat framework, SQOR expression and activity are highly context-dependent: upregulation protects tissues from ischemia/reperfusion injury and ferroptosis, whereas inhibition may be beneficial in chronic heart failure or early cancer by allowing endogenous H_2_S to accumulate. Therefore, SQOR is not only a key molecule for understanding the pathophysiological mechanisms of these complex diseases, but is also increasingly being recognized as a promising therapeutic target and diagnostic biomarker (Lee et al., 2024; Alam et al., 2023; Cai et al., 2024).

This review provides a systematic overview of the core biological functions of SQOR in H_2_S metabolism and explores recent research advances across diseases of the cancer, cardiovascular, nervous, and reproductive systems. By comprehensively elucidating SQOR’s mechanisms of action under different physiological and pathological conditions, we highlight its pivotal role as a “metabolic rheostat” in maintaining sulfur homeostasis and cellular energy metabolism. These insights offer a solid theoretical basis for developing SQOR as a target for disease diagnosis and therapy, and point to future research directions in the field. Table 1 presents SQOR and ferroptosis defense mechanisms: mechanistic insights, controversies, and disease effects dependent on specific contexts. Table 2 illustrates SQOR-Mediated Ferroptosis Defense Across Diseases: Shared Mechanisms, Context-Specific Roles, and Positive/Negative Effects.

**TABLE 1:** SQOR and ferroptosis defense: Mechanistic insights, controversies, and context-dependent disease roles.

Theme/controversy	Representative studies	Key mechanism/point of debate	Notes	References
I. Enzymology and mechanistic insights				
Structural features and catalytic mechanism	Jackson et al. resolved the human SQOR crystal structure and identified a positively charged groove near the catalytic site, capturing the 4α-thiol-flavin intermediate; Landry et al. discovered the unique Cys201–S–S–S–Cys379 trisulfide and demonstrated its importance for catalytic activity; subsequent QM/MM calculations showed the trisulfide lowers the nucleophilic attack barrier by ∼6.3 kcal mol^-1^ and increases catalytic efficiency by ∼10^5^-fold	SQOR catalyzes H_2_S oxidation via a one-step FAD→CoQ electron transfer accompanied by proton movement. The trisulfide is central to high catalytic efficiency; its loss markedly decreases thermal stability	Highlights SQOR’s distinctive catalytic center and electron-transfer mechanism, providing a structural basis for future inhibitor/activator design	Jackson et al. (2019), Landry et al. (2021a), Landry et al. (2021b), Landry et al. (2020)
Physiological sulfur acceptor debate	Landry et al. used membrane-mimetic systems to support GSH as a more effective sulfur acceptor, proposing an H_2_S → GSSH → sulfate route; Augustyn et al. directly measured hepatic/cardiac sulfite, arguing that high sulfite concentrations provide superior catalytic efficiency, thus supporting the “sulfite hypothesis.”	Intense debate remains over whether GSH or sulfite is the dominant acceptor *in vivo*. Landry emphasizes GSH advantage under membrane conditions; Augustyn contends sulfite levels have been underestimated and, when recalculated, catalytic efficiency far exceeds that of GSH.	Underscores the need for accurate microenvironmental measurements and suggests possible coexistence of multiple acceptors depending on context	Landry et al. (2017), Augustyn et al. (2017)
Microenvironmental regulation and evolutionary specialization	Landry et al. noted inner-mitochondrial-membrane lipids can amplify sulfite interference; in periodontitis, methanethiol (MeSH) generates non-metabolizable methyl-polysulfide (MeSSH) that blocks the SQOR pathway; Romanelli-Cedrezz et al. showed in *C. elegans* that SQRD-1 uses UQ or RQ to detoxify H_2_S, whereas SQRD-2 acts as a negative regulator	SQOR activity depends not only on the catalytic core but also on membrane lipids, pseudo-substrates, and species-specific factors. Functional divergence of paralogs in *C. elegans* indicates high evolutionary plasticity	Emphasizes how microenvironment and species adaptation modulate SQOR function and helps interpret disease-specific behavior	Landry et al. (2018), Romanelli-Cedrez et al. (2024)
II. SQOR and Ferroptosis: A New Defense Axis and Its Controversies				
H_2_Se–CoQ_10_H_2_ pathway	Lee et al. demonstrated that SQOR uses H_2_Se to reduce ubiquinone to ubiquinol, rapidly scavenging lipid radicals and establishing a GPX4-independent anti-ferroptosis pathway	SQOR donates electrons via H_2_Se to generate CoQ_10_H_2_, forming an antioxidant barrier parallel to the GPX4–GSH axis. This mechanism is rapid and does not require synthesis of new selenoproteins	Suggests the H_2_Se–SQOR axis can compensate under GPX4 inhibition or GSH depletion and maintain membrane antioxidant capacity	Lee et al. (2024)
Bidirectional roles in tumor contexts	In hypoxic tumors such as PDAC, Lin et al. observed that high SQOR expression elevates CoQ_10_H_2_, enhancing antioxidant capacity and driving tumor growth and drug resistance; conversely, in immune-responsive tumors (e.g., osteosarcoma), Wang et al. found high SQOR strengthens antioxidant defense and immune activation, showing antitumor effects	SQOR can shape tumor fate by tuning CoQ_10_H_2_ and redox defense, yet its direction depends on metabolic needs and immune context—pro-growth in hypoxic/high-demand tumors but suppressive in immune-active tumors	Highlights the need to evaluate tumor metabolic background before SQOR-targeted therapy—avoid one-size-fits-all	Lin et al. (2025), Wang et al. (2024b)
Protective role in kidney injury	Cai et al. showed SQOR deficiency exacerbates cisplatin-induced tubular ferroptosis and mitochondrial damage, while appropriate compensation alleviates tissue injury	By maintaining CoQ_10_H_2_ and removing H_2_S, SQOR protects against oxidative lipid damage in acute renal injury	Demonstrates a protective, non-tumor role of SQOR in ferroptosis control	Cai et al. (2024)

III. Context-dependent and controversial roles across diseases			
Disease/context	Controversy/mechanism	Representative evidence	References
Cardiovascular and renal disorders	On one hand, PC deletion leads to SQOR ubiquitination and degradation, thereby activating cGAS–STING and inducing metabolic reprogramming and renal fibrosis; on the other hand, early diabetic kidney disease may show SQOR upregulation as a compensatory response, which over time could aggravate hypoxic stress (as discussed in the text)	Illustrates SQOR’s double-edged nature in metabolic disease: it can protect via antioxidant effects, yet excessive degradation or maladaptive compensation may drive inflammation and fibrosis	Bushell et al. (2023), Huang et al. (2025)
Intestinal inflammation and cancer	In adult IBD, SQOR is markedly downregulated and independent of inflammation severity, suggesting an intrinsic risk factor; in UC models, SQOR loss enhances Drp1 activity, suppresses PGC1α, and disrupts the barrier. In CRC, SQOR is generally downregulated (H_2_S accumulation ETC., activation). However, Dawoud et al. reported early upregulation is protective, whereas late downregulation promotes metastasis, proposing the “respiratory shield” hypothesis	These findings indicate stage-dependent shifts in SQOR’s role across IBD and CRC, calling for stage-tailored interventions	Piran et al. (2021), Dawoud et al. (2024), Stummer et al. (2022), Ma et al. (2020)
Bone metabolism and reproductive health	Changes in SQOR expression affect BAX/BCL-2 balance, energy metabolism, and oxidative stress in osteonecrosis/osteoporosis; in male reproduction, ASB1-mediated ubiquitination maintains SQOR stability—ASB1 loss causes SQOR accumulation and H_2_S depletion, leading to sperm defects	These mechanisms reveal how SQOR shapes mitochondrial homeostasis in bone and reproductive systems; the revised table emphasizes molecular regulation rather than merely listing phenotypes	Cai et al. (2018), Ma et al. (2024), Lv et al. (2025)

Abbreviations: CoQ, Coenzyme Q; CoQ10 (UQ), Coenzyme Q10/Ubiquinone; CoQ10H2 (UQH2), Ubiquinol; CRC, colorectal cancer; DHODH, dihydroorotate dehydrogenase; ETC.,, electron transport chain; FAD, flavin adenine dinucleotide; FSP1 (AIFM2), Ferroptosis Suppressor Protein 1; GPX4 Glutathione Peroxidase 4; GSH, glutathione; H_2_Se, Hydrogen Selenide; I/R, Ischemia–Reperfusion; IBD, inflammatory bowel disease; IMM, inner mitochondrial membrane; MeSH, methanethiol; MeSSH, methyl persulfide; NAD(P)H, Nicotinamide Adenine Dinucleotide (Phosphate), Reduced; PDAC, pancreatic ductal adenocarcinoma; PL-OH, phospholipid alcohol; PL-OOH, phospholipid hydroperoxide; PMID, PubMed Identifier; RQ, rhodoquinone; SQOR; sulfide quinone oxidoreductase; UQ, ubiquinone; UQH2, ubiquinol.

**TABLE 2:** SQOR-Mediated Ferroptosis Defense Across Diseases: Shared Mechanisms, Context-Specific Roles, and Positive/Negative Effects SQOR‐mediated ferroptosis defence and context-specific roles.

Condition (model)	SQOR role/phenotype	Mechanism(s)	Unique aspects/notes	References
Alternative ferroptosis regulation beyond GPX4	SQOR uses hydrogen selenide (H_2_Se) to reduce ubiquinone (CoQ_10_) to the antioxidant ubiquinol (CoQ_10_H_2_), quenching lipid peroxides and establishing a GPX4-independent ferroptosis defence	H_2_Se→SQOR→CoQ_10_H_2_ axis scavenges lipid peroxides; complements or compensates for GPX4–GSH/FSP1	Establishes a selenium-dependent ferroptosis defence pathway distinct from canonical GPX4; clarifies that not all lipid peroxides require GPX4 for detoxification	Lee et al. (2024)
Hypoxic tumours (e.g., pancreatic cancer)	High SQOR expression promotes tumour growth and drug resistance	Increases CoQ_10_H_2_ pool and antioxidant capacity under hypoxia, enhancing resistance to ferroptotic stress	Illustrates that SQOR can be protumoural when oxygen is limiting, contrasting with its protective roles elsewhere	Lin et al. (2025)
Immune-sensitive tumours (e.g., osteosarcoma)	High SQOR expression enhances antioxidant defence and immune activation, exerting antitumour effects opposite to hypoxic tumours	Boosts CoQ_10_H_2_ production, supports immune responses, and limits ferroptosis	Demonstrates context-dependent outcomes; SQOR can be tumour-suppressive in immune-competent settings	Wang et al. (2024b)
Renal injury and cisplatin-induced ferroptosis	SQOR deficiency exacerbates cisplatin-induced ferroptosis and mitochondrial damage; functional compensation alleviates injury	Loss of SQOR diminishes CoQ_10_H_2_ regeneration and increases lipid peroxidation	Highlights SQOR as a therapeutic target for protecting kidneys against ferroptotic damage during chemotherapy	Cai et al. (2024)

SQOR in neurological diseases				
Disease/condition	SQOR role/phenotyp	Mechanism(s)	Unique aspects/notes	References
Leigh syndrome	SQOR loss-of-function mutations cause severe neurodegeneration	SQOR deletion leads to H_2_S accumulation, inhibits Complex IV of the electron transport chain and impairs energy metabolism	Pathogenic variants in SQOR are a treatable cause of Leigh-like syndrome	Landry et al. (2021a)
Ischaemic brain injury	SQOR over-expression or peptide mimetic SS20 is neuroprotective	Enhanced H_2_S detoxification increases ATP generation, improves mitochondrial function and reduces infarct volume	Synthetic mimetic SS20 activates SQOR and mimics its protective effect	Kanemaru and Ichinose (2025)
Amyotrophic lateral sclerosis (C9orf72 model)	Pathogenic GA50 dipeptide inhibits SQOR, triggering inflammation; small molecule irigenin rescues function	GA50 binds SQOR and blocks its activity, leading to NLRP3 inflammasome activation; irigenin disrupts GA50–SQOR interaction	First evidence that dipeptide repeats can target SQOR; offers a potential therapy via small-molecule modulators	Fu et al. (2023)
SQOR in cardiovascular diseases				
Aortic valve stenosis	SQOR activation suppresses valvular fibrosis and calcification	Hydrogen sulfide activates the NRF2–SQOR pathway, increasing antioxidant defences	Demonstrates SQOR as a mediator of hydrogen sulfide signalling in valve pathology	Song et al. (2024)
Chronic heart failure	Pharmacological inhibition of SQOR (STI-1) relieves myocardial hypertrophy and dysfunction	Inhibiting SQOR elevates endogenous H_2_S levels, counteracting heart failure	Represents the first SQOR inhibitor; suggests that raising H_2_S via SQOR inhibition can be therapeutic	Jackson et al. (2022)
Myocardial ischaemia–reperfusion injury	Augmenting SQOR activity protects against I/R injury	Persulfide donors activate SQOR, reduce infarct size and maintain energy metabolism	Shows that boosting persulfide supply enhances SQOR-mediated protection	Nishimura et al. (2025)
Vascular endothelial homeostasis	SQOR expression is essential for endothelial H_2_S clearance and mitochondrial respiratory stability	Regulates endothelial H_2_S metabolism, maintaining redox balance and mitochondrial function	Emphasises that endothelial health depends on adequate SQOR levels	Star et al. (2023)
SQOR in renal diseases				
Diabetic kidney disease (early)	SQOR upregulation may compensate for H_2_S deficiency but can exacerbate hypoxic injury	Increased SQOR activity attempts to clear excess sulfide; in hypoxia this may worsen oxidative stress	Suggests a dual role: compensatory yet potentially harmful in advanced DKD	Bushell et al. (2023)
CoQ deficiency	CoQ shortage causes SQOR degradation, blocking H_2_S metabolism and aggravating oxidative injury	Without sufficient CoQ, SQOR is destabilised and proteolysed; H_2_S accumulates	Highlights the dependence of SQOR stability on CoQ availability	Kleiner et al. (2018)
Renal fibrosis	Loss of pyruvate carboxylase leads to SQOR ubiquitination and degradation, driving fibrosis	PC deletion → SQOR ubiquitination → decreased H_2_S oxidation → cGAS–STING activation and metabolic reprogramming	Connects SQOR to innate immunity and fibrosis via the cGAS–STING pathway	Huang et al. (2025)
SQOR in gastrointestinal diseases and colorectal cancer				
Inflammatory bowel disease (age-related)	SQOR is markedly downregulated in adult IBD, independent of inflammation severity	Decreased H_2_S detoxification may predispose to oxidative stress and tissue injury	Suggests SQOR downregulation as an independent risk factor for adult IBD	Stummer et al. (2022)
Ulcerative colitis	SQOR loss exacerbates colitis by disrupting mitochondrial dynamics and antioxidant defences	Loss of SQOR increases Drp1 activity, suppresses PGC1α and weakens gut barrier integrity	Links SQOR deficiency to altered mitochondrial fission–fusion and epithelial barrier disruption	Ma et al. (2020)
Colorectal cancer (primary/metastatic)	SQOR downregulation leads to H_2_S accumulation, enhanced electron transport and tumour growth	H_2_S buildup supports mitochondrial respiration and proliferation	Suggests SQOR as a tumour suppressor in CRC; loss promotes growth	Piran et al. (2021)
Colorectal cancer (biphasic expression)	Early SQOR upregulation protects cells, while late downregulation promotes metastasis	Biphasic expression may create a ‘respiratory shield’ enabling adaptation to oxidative stress	Emphasises stage-dependent roles of SQOR in cancer progression	Dawoud et al. (2024)
Colorectal cancer (NAC treatment)	N-acetylcysteine (NAC) induces SQOR, enhancing H_2_S metabolism and antioxidant defences	NAC increases persulfide production via MST, thereby up-regulating SQOR and boosting antioxidant capacity	Demonstrates a therapeutic strategy to modulate SQOR through thiol supplementation	Zuhra et al. (2019)
SQOR in bone metabolism and reproductive health				
Postmenopausal osteoporosis	A genetic polymorphism in SQRDL (C allele of rs1044032) is protective	Allele may influence SQOR expression or activity, conferring resistance to bone loss	Provides evidence for a genetic modifier of SQOR in osteoporosis	Cai et al. (2018)
Femoral head necrosis (SONFH)	SQOR upregulation is associated with BAX/BCL-2 imbalance, oxidative stress and energy dysregulation	Excess SQOR may disrupt apoptosis signalling and cellular energy metabolism in bone tissue	Highlights a potential pathological over-expression of SQOR in bone necrosis	Ma et al. (2024)
Male infertility	Balanced SQOR turnover is essential for spermatogenesis; ASB1 loss leads to SQOR accumulation, H_2_S depletion and oxidative stress	ASB1 mediates SQOR ubiquitination; its loss → SQOR build-up → decreased H_2_S → oxidative damage → sperm defects	Underlines the importance of SQOR homeostasis for male fertility	Lv et al. (2025)

Abbreviations: SQOR, Sulfide:quinone oxidoreductase (gene symbol SQRDL); SQRDL, Sulfide quinone reductase–like; H_2_Se—Hydrogen selenide; H_2_S—Hydrogen sulfide; CoQ/CoQ_10_ — Coenzyme Q10/Ubiquinone; CoQ_10_H_2_ — Ubiquinol (reduced CoQ10); GPX4 — Glutathione peroxidase 4; GSH, Glutathione; FSP1 (AIFM2) — Ferroptosis suppressor protein 1; NAD(P)H—Reduced nicotinamide adenine dinucleotide (phosphate); I/R—Ischemia–reperfusion; NRF2 — Nuclear factor erythroid 2–related factor 2; DKD, Diabetic kidney disease; PC, Pyruvate carboxylase; cGAS–STING, Cyclic GMP-AMP, synthase–Stimulator of Interferon Genes; Drp1 — Dynamin-related protein 1; PGC1α—Peroxisome proliferator-activated receptor gamma coactivator 1-alpha; CRC, Colorectal cancer; MST, Mercaptopyruvate sulfurtransferase; SONFH, Steroid-induced osteonecrosis of the femoral head; BAX—BCL-2–associated X protein; BCL-2 — B-cell lymphoma 2; ASB1 — Ankyrin repeat and SOCS, box protein 1; ALS, Amyotrophic lateral sclerosis; C9orf72 — Chromosome 9 open reading frame 72; GA50 — Poly-GA, dipeptide (length 50); NLRP3 — NLR, family pyrin domain–containing 3; SS-20, Mitochondria-targeted peptide SS-20; STI-1 — SQOR, inhibitor (pharmacological tool); ATP, Adenosine triphosphate; Complex IV, Cytochrome c oxidase; NAC—N-acetylcysteine.

## Enzyme structure and catalytic mechanism of SQOR

### Structural biology breakthrough: discovery of a unique catalytic core

SQOR structural biology has achieved a milestone breakthrough (Jackson et al., 2019). Analyzed the 2.59 Å crystal structure of human SQOR and found that the enzyme is monomeric with a positively charged surface groove capable of binding receptor molecules such as sulfite and glutathione. This groove connects to the catalytic cysteine (Cys379) via a short channel, explaining the structural basis for SQOR’s adaptability to multiple substrates. Jackson and colleagues also captured the key intermediate 4a-thiolflavin adduct in the active site, showing that electrons are transferred from the flavin cofactor (FAD) to CoQ in a single ∼3.7 Å jump, with proton transfer mediated by residues Trp345 and Ser378. These findings refined the mechanistic understanding of H_2_S oxidation.

**FIGURE 2:**
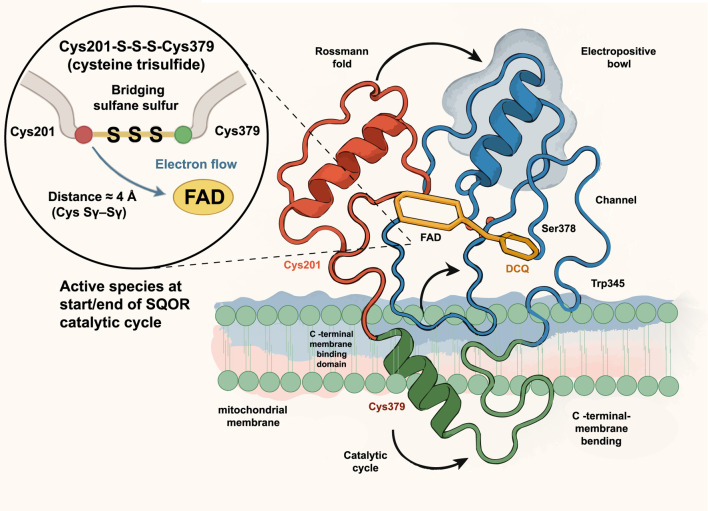
Crystal structure of human SQOR. The ribbon model shows SQOR anchored to the mitochondrial membrane through a C-terminal membrane-binding helix and organized into a Rossmann fold (red) and an electropositive bowl with an access channel (blue). The active site contains the cofactor flavin adenine dinucleotide (FAD, yellow) and a decyl-coenzyme Q analog (DCQ, orange) near key channel residues Ser378 and Trp345. A magnified inset depicts the unique cysteine trisulfide cofactor, Cys201–S–S–S–Cys379, in which the bridging sulfane sulfur links Cys201 and Cys379 (distance ≈3.7 Å) and directs electron flow to FAD; this trisulfide constitutes the active species at the start and end of the SQOR catalytic cycle. FAD, flavin adenine dinucleotide; DCQ, decyl coenzyme Q analog; Cys201/Cys379, cysteine residues 201 and 379; Ser378, serine residue 378; Trp345, tryptophan residue 345.

Subsequently, Landry et al. discovered that SQOR’s active site is not a traditional cysteine disulfide, but a unique “trisulfide bond” linking Cys201 and Cys379 via an additional sulfur atom (Cys201–S–S–S–Cys379). This is the first such structure identified in the flavin disulfide reductase family (Landry et al., 2021a; Landry et al., 2021b). The authors used cyanide to specifically cleave the trisulfide bond and remove the central sulfur, which caused a complete loss of catalytic activity. When H_2_S was added, the trisulfide bond spontaneously reformed and enzyme activity was restored. This reversible “disassembly–reconstruction” experiment demonstrated convincingly that the cysteine trisulfide bond is an essential cofactor for SQOR catalysis. Further quantum mechanics/molecular mechanics (QM/MM) calculations showed that the trisulfide cofactor lowers the energy barrier for nucleophilic attack by H_2_S by ∼6.3 kcal/mol, increasing catalytic efficiency by approximately 105-fold. Consistent with this, loss of the trisulfide bond sharply reduced SQOR’s thermal denaturation temperature from 66 C to ∼36.5 C (Landry et al., 2020). These findings establish the cysteine trisulfide as the highly efficient catalytic core of SQOR, revolutionizing our understanding of how this enzyme operates. Notably, the trisulfide cofactor not only enhances catalytic efficiency but also facilitates electron transfer from sulfide or selenide to ubiquinone, providing a structural basis for SQOR’s role as a metabolic rheostat. Figure 2 shows the crystal structure of human SQOR.

### The debate over physiological sulfur receptors: glutathione or sulfite?

After unveiling SQOR’s unusual active-site structure, a key question in its catalytic cycle gained attention: under physiological conditions, to which acceptor molecule does the sulfur atom from the enzyme’s persulfide intermediate (Cys–SSH) preferentially transfer? Two camps have formed on this issue: the “glutathione (GSH) hypothesis” and the “sulfite hypothesis.” Landry et al. conducted experiments with SQOR embedded in a membrane-mimicking nanoplate system to simulate the inner mitochondrial membrane environment. Their results supported the GSH hypothesis: SQOR in a membrane environment had significantly higher catalytic efficiency for H_2_S than in detergent, and sulfur transfer from the enzyme to its receptor was the rate-limiting step of the entire cycle. Given that intracellular GSH is in the millimolar range (much higher than sulfite’s micromolar range), their modeling showed that the overall reaction rate using GSH as the sulfur acceptor is about five times higher than with sulfite. They concluded that GSH is the primary sulfur receptor of SQOR *in vivo*, and proposed a simplified pathway in which SQOR oxidizes H_2_S to generate glutathione persulfide (GSSH), which is then converted to sulfite and sulfate—thus avoiding the need for sulfite to directly cross membranes (Landry et al., 2017). In contrast (Augustyn et al., 2017), directly measured sulfite levels in rat liver and heart and found actual concentrations (∼9.2 μM in liver, ∼38 μM in heart) nearly two orders of magnitude higher than previously assumed. Plugging these values into kinetic models reversed the conclusion: sulfite as the acceptor yielded ∼500-fold higher catalytic efficiency than GSH, accounting for roughly 62% and 92% of H_2_S oxidation flux in liver and heart, respectively. They thus put forward the sulfite hypothesis, arguing that sulfite is the primary physiological sulfur acceptor for SQOR in the liver and heart—providing strong *in vivo* support for the classic Jorns model. This lively debate underscores the importance of accurate parameters when extrapolating *in vitro* enzyme data: the two hypotheses are not mutually exclusive, but remind us that we must directly and precisely quantify substrate concentrations in the mitochondrial matrix when constructing physiological models. In the future, developing technologies for real-time monitoring of metabolite levels in specific cellular compartments will be essential to finally reveal which sulfur acceptor predominates in the SQOR catalytic cycle.

### Microenvironment control and dynamic characteristics

Studies also show that SQOR activity is finely tuned by its microenvironment and substrate status. For example, the lipid milieu of the inner mitochondrial membrane itself modulates SQOR—it prevents non-productive binding of GSH while enhancing sulfite’s interference effect, together with substrate concentrations ensuring that H_2_S is oxidized preferentially and efficiently. In certain pathological conditions (such as periodontitis), high levels of methyl mercaptan (MeSH) act as a competitive false substrate for SQOR, reacting with the enzyme to form persulfide products (MeSSH) that cannot be further metabolized. This effectively blocks the normal sulfur oxidation pathway and disrupts sulfur homeostasis (Landry et al., 2018). Moreover, over the course of evolution, SQOR function has specialized in different species: in the nematode *Caenorhabditis elegans*, SQOR family members have diverged such that SQRD-1 serves as the primary H_2_S detoxification enzyme (able to use either ubiquinone or rhodoquinone as electron acceptor), whereas SQRD-2 acts as a negative regulator that accelerates recovery from H_2_S exposure (Romanelli-Cedrez et al., 2024). These findings illustrate SQOR’s dynamic nature—its activity, efficiency, and even substrate preference can change with cellular metabolic status (e.g., selenium availability), membrane lipid composition, pathological factors (e.g., competing substrates), and species-specific evolutionary pressures. This high degree of functional flexibility poses challenges for therapeutic exploitation: when designing SQOR-targeted interventions, one must consider that SQOR may have diametrically opposite effects in different contexts to avoid unintended disruptions of the broader sulfur metabolic network.

### SQOR and ferroptosis

Ferroptosis is an iron-dependent form of programmed cell death driven by the accumulation of iron-catalyzed lipid peroxides in cell membranes, which causes lethal membrane damage (Tang and Kroemer, 2020). Typically, when cellular antioxidant defenses are compromised, unchecked lipid peroxides trigger ferroptosis (Su et al., 2020). Glutathione peroxidase 4 (GPX4) is a well-known enzyme that protects against ferroptosis, as it uses glutathione (GSH) to reduce lipid peroxides to fatty alcohols and thereby terminates lipid peroxidation chain reactions. However, GPX4 is not the only ferroptosis defense. When ferroptosis threatens, cells deploy multiple antioxidant defense systems to halt lipid-peroxidation chain reactions. Beyond the cysteine/GSH/GPX4 axis—in which system Xc^−^ imports cystine, fuels glutathione biosynthesis, and GPX4 reduces phospholipid hydroperoxides to lipid alcohols (Weaver and Skouta, 2022)—cells also use coenzyme Q_10_ (CoQ_10_) and its reduced form ubiquinol (CoQ_10_H_2_) as lipophilic radical-trapping agents. Myristoylated FSP1 localizes to the plasma membrane and, in an NAD(P)H-dependent manner, reduces oxidized CoQ_10_ to CoQ_10_H_2_, establishing a defense pathway parallel to GPX4 (Bersuker et al., 2019). Notably, the anti-ferroptotic activity of FSP1 does not alter glutathione levels, indicating a GPX4-independent mechanism. In addition, the inner-mitochondrial-membrane enzyme dihydroorotate dehydrogenase (DHODH) transfers electrons derived from pyrimidine synthesis to ubiquinone, reducing it to ubiquinol and thereby suppressing ferroptosis in parallel with mitochondrial GPX4 (Mao et al., 2021).

Recent work shows that the inner-membrane enzyme sulfide:quinone oxidoreductase (SQOR) does more than oxidize H_2_S—it can also function as a ubiquinone reductase. When cells reduce inorganic selenium (e.g., selenite) taken up via the cystine/glutamate antiporter xCT (SLC7A11) to hydrogen selenide (H_2_Se), SQOR uses H_2_Se as an electron donor to rapidly reduce ubiquinone to ubiquinol, conferring high-efficiency radical-trapping capacity within the mitochondrial inner membrane (Lee et al., 2024). This process is independent of *de novo* selenoprotein synthesis; thus, even when GPX4 translation is limited, SQOR can rapidly provide antioxidant protection. Genetic or pharmacologic ablation of SQOR enhances mitochondrial lipid peroxidation triggered by GPX4 inhibitors, whereas SQOR overexpression partially restrains ferroptosis. Because SQOR acts within mitochondria, its anti-ferroptotic effects are compartmentalized, complementing plasma-membrane FSP1 and cytosolic/mitochondrial GPX4. Taken together, the SQOR–H_2_Se–CoQ_10_H_2_ pathway constitutes an independent yet complementary branch that affords partial compensation when the GPX4–GSH axis is compromised, but cannot fully substitute for GPX4 or FSP1.

At the same time, SQOR’s effect on ferroptosis sensitivity is highly context-dependent and bidirectional. For example, in hypoxic tumors such as pancreatic cancer (Lin et al., 2025), high SQOR expression increases mitochondrial CoQ_10_H_2_ levels, boosting the cells’ antioxidant capacity and anti-ferroptotic defenses, thereby making the cancer cells more resistant to ferroptosis-induced oxidative stress (promoting tumor growth and drug resistance). In contrast, in immunoresponsive tumors such as osteosarcoma, high SQOR expression enhances systemic antioxidant homeostasis and anti-tumor immune activity, thus inhibiting tumor progression (suppressing tumor growth) (Wang Y. et al., 2024). Therefore, SQOR’s influence on ferroptosis in cancer depends on tumor metabolic demands and the immune microenvironment: it may help tumor cells survive by preventing ferroptosis, or alternatively enhance anti-tumor immunity by permitting some oxidative damage. These sharply divergent effects indicate that SQOR’s role is highly situation-dependent, so any SQOR-targeted therapy must be tailored to context rather than “one size fits all”. Furthermore, before considering SQOR-based therapies, it will be necessary to develop companion diagnostic tools to assess SQOR activity in a patient’s tissues and determine how dependent cell survival is on SQOR, thereby guiding whether to inhibit or activate SQOR to achieve the desired therapeutic outcome.

Notably, SQOR’s protective effect against ferroptosis is also evident outside of cancer. For example, in a cisplatin-induced acute kidney injury model, SQOR deficiency exacerbated mitochondrial dysfunction and lipid peroxidation in renal tubular cells, significantly increasing ferroptotic cell death and tissue damage; conversely, normal SQOR activity eliminated excess sulfide and preserved CoQ_10_H_2_’s antioxidant function, thereby mitigating cisplatin-induced oxidative damage and protecting the kidney (Cai et al., 2024). These studies demonstrate that SQOR can either promote or inhibit ferroptosis depending on the biological context.

In aggregate, by channeling H_2_S/selenium metabolism into ubiquinol production, SQOR forms the third arm of the ferroptosis-defense network alongside the GPX4–GSH and FSP1–CoQ_10_ axes. Its independent, complementary, and partially compensatory nature implies a layered cellular strategy against ferroptosis, with the impact of SQOR governed by H_2_S/H_2_Se availability and mitochondrial status in the microenvironment (Lee et al., 2024). Accordingly, SQOR is an attractive therapeutic target, yet its modulation should be precisely matched to tumor metabolic and immune contexts; rigorous clinical investigation remains essential.

### Neurological diseases

As a “gatekeeper” of sulfur metabolism, SQOR ensures that H_2_S remains in a beneficial (signaling) range rather than a toxic one. If SQOR activity declines or its regulation fails, H_2_S shifts from a signaling molecule to a cytotoxin, leading to mitochondrial dysfunction, decreased ATP production, and heightened inflammation. This cascade is conserved across many neurological disorders. Numerous studies have shown that a common pathological feature in many neurodegenerative diseases is reduced SQOR expression. SQOR deficiency causes H_2_S to accumulate and potently inhibit cytochrome c oxidase (Complex IV), resulting in a state of tissue hypoxia and metabolic crisis in the brain. This has been confirmed in mice with SQOR gene defects: loss of SQOR function leads to a fatal Leigh-like syndrome (Landry et al., 2021a; Landry et al., 2021b), due to H_2_S toxicity accumulation and respiratory chain dysfunction (Kanemaru et al., 2024). Conversely, increasing SQOR levels in the brain can clear excess H_2_S and sustain ATP production, thereby improving the brain’s tolerance to hypoxia/ischemia. For instance, neuron-specific overexpression of SQOR significantly reduced cerebral infarct size and delayed energy depletion in ischemic brain injury models (Marutani et al., 2021; Kanemaru and Ichinose, 2025).

In addition, SQOR dysfunction is closely linked to neuroinflammatory processes. In ischemic stroke, downregulation of SQOR is associated with exacerbated inflammatory responses in the brain. In a model of amyotrophic lateral sclerosis (ALS) caused by C9orf72 gene mutations, the pathogenic poly-dipeptide GA50 directly binds to and inhibits SQOR, leading to excessive NLRP3 inflammasome activation in microglia and consequent mitochondrial dysfunction (Fu et al., 2023). Given the crucial role of SQOR in these neuropathological mechanisms, targeting the SQOR pathway represents a promising neuroprotective strategy.

**FIGURE 3:**
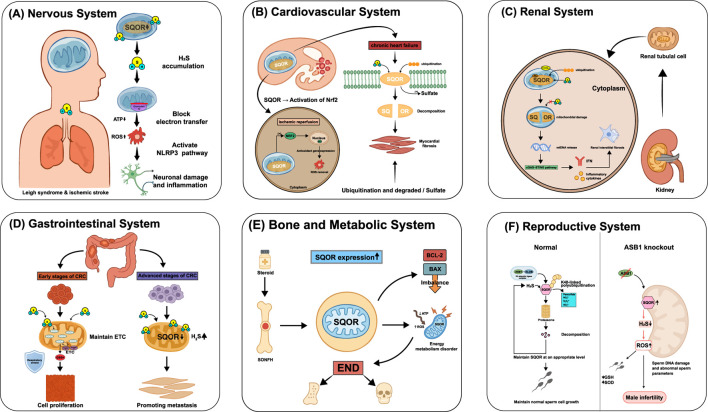
Role of SQOR in diseases across systems. **(A)** Nervous system: SQOR deficiency or mutation causes H_2_S accumulation, blocks mitochondrial electron transfer, decreases adenosine triphosphate (ATP), elevates reactive oxygen species (ROS), activates the NOD-like receptor thermal protein domain-associated protein 3 (NLRP3) inflammasome and leads to neuronal damage and inflammation, as in Leigh syndrome and ischemic stroke. **(B)** Cardiovascular system: during ischemia-reperfusion, SQOR activation enhances nuclear factor erythroid 2-related factor 2 (Nrf2)-dependent antioxidant gene expression and ROS clearance; in chronic heart failure, SQOR is ubiquitinated and degraded, leading to excess H_2_S and myocardial fibrosis. **(C)** Renal system: loss of SQOR in renal tubular cells causes mitochondrial damage, release of mitochondrial DNA (mtDNA) and activation of the cyclic GMP-AMP synthase-stimulator of interferon genes (cGAS-STING) pathway, inducing interferon (IFN) production and renal interstitial fibrosis. **(D)** Gastrointestinal system: in early colorectal cancer (CRC), SQOR maintains electron transport chain (ETC.) function and supports epithelial barrier integrity, whereas in advanced CRC, SQOR downregulation and increased H_2_S promote metastasis. **(E)** Bone and metabolic system: in steroid-induced osteonecrosis of the femoral head (SONFH), upregulated SQOR causes energy metabolism disorder (ATP↓, ROS↑) and an imbalance between B-cell lymphoma 2 (BCL-2) and BCL-2-associated X protein (BAX), contributing to osteonecrosis. **(F)** Reproductive system: under normal conditions, ankyrin repeat and SOCS box protein 1 (ASB1)-mediated K48-linked polyubiquitination maintains SQOR at appropriate levels to support normal sperm development; ASB1 knockout increases SQOR, elevates H_2_S and ROS, damages sperm DNA and leads to male infertility. SQOR, sulfide:quinone oxidoreductase; H_2_S, hydrogen sulfide; ATP, adenosine triphosphate; ROS, reactive oxygen species; NLRP3, NOD-like receptor thermal protein domain-associated protein 3; Nrf2, nuclear factor erythroid 2-related factor 2; mtDNA, mitochondrial deoxyribonucleic acid; cGAS-STING, cyclic GMP-AMP synthase-stimulator of interferon genes; IFN, interferon; CRC, colorectal cancer; ETC., electron transport chain; SONFH, steroid-induced osteonecrosis of the femoral head; BCL-2, B-cell lymphoma 2; BAX, B-cell lymphoma-2-associated X protein; ASB1, ankyrin repeat and SOCS box protein 1; GSH, glutathione; SOD, superoxide dismutase.

A growing body of preclinical work supports this strategy: boosting SQOR activity or reducing H_2_S can protect the nervous system. Neuron-specific SQOR overexpression has been shown to decrease the volume of cerebral infarction after stroke (Marutani et al., 2021; Kanemaru and Ichinose, 2025). Scavenging H_2_S with hydroxocobalamin protects mitochondrial function in ischemic brain tissue by preventing H_2_S from inhibiting Complex IV (Friederich et al., 2020). A synthetic SQOR analog, SS20, efficiently scavenges H_2_S and generates thiolate anions, significantly alleviating ischemic brain injury in mice (Kanemaru and Ichinose, 2025). Supplementing sulfane sulfur donors such as glutathione trisulfide (GSSSG) also provided neuroprotection: in models of Parkinson’s disease, spinal cord ischemia, and chemotherapy-induced peripheral neuropathy, GSSSG improved functional outcomes, with intranasal delivery showing good potential (Ichinose et al., 2024). For Leigh syndrome due to SQOR genetic defects, reducing endogenous H_2_S production has proven beneficial: for example, oral metronidazole (to eliminate H_2_S-producing gut microbes) or a low-sulfur diet reversed neuro-metabolic abnormalities in SQOR-deficient mice (Kanemaru et al., 2024). Moreover, blocking pathological interactions involving SQOR is a novel approach–the natural compound iridoside can disrupt GA50’s binding to SQOR, thereby inhibiting NLRP3 inflammasome activation in ALS models (Fu et al., 2023). Although these SQOR-targeted interventions have shown significant neuroprotective effects in animal models, their efficacy and safety in humans remain to be established through further research. Figure 3 illustrates the diverse roles of SQOR in several systemic diseases. In the nervous system, the key mechanisms and outcomes are summarized in Figure 3A.

### Cardiovascular disease

In the heart and vasculature, SQOR determines the rate of H_2_S oxidation, whereas H_2_S itself confers protection through vasodilatory, anti-inflammatory, and antioxidant effects. The two factors engage in a dynamic balance: in acute injury, enhancing SQOR helps maintain mitochondrial respiration and clear excess sulfur; in chronic conditions of H_2_S deficiency, excessive SQOR activity may undermine the heart’s intrinsic protective mechanisms. Thus, SQOR has a dual role in cardiovascular disease and can exert opposite effects under different pathological conditions. These context-dependent effects in acute ischemia–reperfusion versus chronic heart failure are depicted in Figure 3B.

On the one hand, SQOR activation appears beneficial in ischemic, hypoxic injury (Song et al., 2024). found that in aortic valve stenosis, exogenous H_2_S activates the NRF2–SQOR pathway, upregulating SQOR and NRF2 and inducing autophagy as well as antioxidant genes (e.g., *NQO1*, *CSE*). These changes inhibited fibrosis, inflammation, and calcification in valvular interstitial cells. On the other hand, inhibiting SQOR in chronic heart failure (where H_2_S signaling is low) has been shown to be cardioprotective. For example, STI1, a highly selective small-molecule SQOR inhibitor developed by Jackson et al. (2022) competitively binds the CoQ binding site of SQOR. In a mouse model of pressure-overload heart failure, STI1 treatment significantly reduced myocardial hypertrophy and fibrosis and improved cardiac function. The presumed mechanism is that suppressing H_2_S catabolism raises endogenous H_2_S levels, thereby activating cardioprotective signaling pathways such as protein sulfhydration. Additionally, other studies have highlighted SQOR’s importance in maintaining cardiovascular mitochondrial function. Nishimura et al. (2025) used plasma technology to generate novel “superhydride” donors that specifically upregulated SQOR in cardiomyocytes. In an ischemia–reperfusion injury model, this SQOR induction preserved mitochondrial energy homeostasis by promoting the conversion of H_2_S to superhydrides, significantly reducing myocardial infarct size. Star et al. (2023) were the first to demonstrate functional SQOR expression in human vascular endothelial cells: only endothelial cells expressing SQOR could efficiently oxidize and remove H_2_S from the circulation, and SQOR activity in these cells was closely tied to mitochondrial respiratory function.

In summary, SQOR can either protect the cardiovascular system by enhancing mitochondrial function and antioxidant capacity, or exacerbate chronic cardiac pathology by depleting H_2_S signaling. Therapeutic strategies targeting SQOR must therefore be calibrated to the disease context. In conditions with low H_2_S signaling (such as heart failure with reduced ejection fraction), SQOR inhibition may be beneficial to restore H_2_S levels, whereas in conditions primarily involving mitochondrial dysfunction (such as ischemia–reperfusion injury), SQOR activation may be advantageous to support mitochondrial function.

### Renal disease

SQOR dysfunction has emerged as an important pathogenic factor in many kidney diseases, with wide-ranging and complex effects. Loss of normal SQOR function directly disrupts hydrogen sulfide (H_2_S) homeostasis, leading to mitochondrial dysfunction, and also exacerbates redox imbalance in the kidney, triggers innate immune activation, and reshapes cellular metabolism (for example, by affecting protein stability and causing excessive ubiquitin-mediated degradation). Collectively, these disturbances drive the progression of renal injury and fibrosis, underscoring SQOR as a central hub in renal pathophysiology. In early diabetic kidney disease, Bushell et al. (2023) observed a significant compensatory increase in SQOR expression in mouse kidneys, suggesting that this response exacerbates local H_2_S deficiency and may aggravate metabolic damage in the hypoxic renal microenvironment. In contrast, in a hereditary nephrotic syndrome caused by coenzyme Q_10_ (CoQ) deficiency, CoQ shortage leads to reduced SQOR stability and rapid SQOR degradation, so that H_2_S cannot be properly cleared. The resulting H_2_S accumulation impairs short-chain fatty acid oxidation, depletes glutathione, and worsens oxidative stress, ultimately damaging renal tubular cells (Kleiner et al., 2018; Qu et al., 2017). Furthermore, abnormal SQOR degradation has been directly implicated in renal fibrosis: Huang et al. (2025) reported that deleting pyruvate carboxylase (PC) in renal tubular epithelial cells destabilizes SQOR, leading to its excessive ubiquitination by E3 ligases and proteasomal degradation. The loss of SQOR then induces mitochondrial damage and mtDNA release, activates the cGAS–STING innate immune pathway, and causes glycolytic reprogramming and excessive extracellular matrix deposition, thereby promoting renal fibrosis. The SQOR destabilization–mtDNA release–cGAS–STING fibrotic cascade is shown in Figure 3C.

Together, these findings indicate that SQOR is a key molecular link between metabolic disturbance, redox imbalance, and immune activation in the kidney. Correcting SQOR dysfunction—whether by tempering its overactivity or supplying missing cofactors—is expected to be a novel therapeutic approach for kidney disease.

### Gastrointestinal disease

In the intestine, SQOR plays a pivotal role in H_2_S oxidation, linking energy metabolism, redox balance, and cellular signaling. Recent studies have revealed complex, multidimensional functions of SQOR in gastrointestinal diseases, especially colorectal cancer (CRC) and inflammatory bowel disease (IBD). Stage-specific changes in CRC and detoxification defects in IBD are organized in Figure 3D.

In CRC, SQOR expression and function show significant stage specificity and duality. Piran et al. (2021) applied a shortest-path analysis to the CRC protein–protein interaction network and found that SQOR is universally downregulated in primary and metastatic CRC. This SQOR suppression impairs sulfide oxidation, leading to intracellular H_2_S accumulation. Under hypoxic conditions, accumulated H_2_S can enhance mitochondrial electron transport chain activity and ATP production, providing an energy boost for rapidly proliferating cancer cells, independent of the Warburg effect. Consistently, SQOR levels show a marked decrease from normal tissue to primary tumors to metastases. Dawoud et al. (2024) further proposed a new concept of “H_2_S metabolic balance” in cancer: during early tumorigenesis, oncogenic mutations may induce a compensatory upregulation of H_2_S-degrading enzymes like SQOR to protect cells from H_2_S toxicity; however, in later cancer stages, those mutations drive persistent downregulation of SQOR, resulting in excess H_2_S accumulation that promotes tumor growth and metastasis. They also described a “respiratory shield” function of SQOR in CRC: by oxidizing H_2_S to glutathione persulfide (GSSH), SQOR sustains normal mitochondrial electron transport, protects cancer cells from H_2_S toxicity, and supports tumor cell proliferation. This pattern of early SQOR elevation and later reduction provides a mechanistic explanation for the biphasic effect of H_2_S in cancer (where low H_2_S concentrations promote tumorigenesis, but high concentrations suppress it). In addition, Scheller et al. (2022) observed that SQOR expression is significantly upregulated when Caco-2 cells spontaneously differentiate into an intestinal epithelial phenotype, suggesting that SQOR helps maintain metabolic homeostasis in normal intestinal cells by enhancing H_2_S detoxification. In CRC, however, aberrant overexpression of SQOR may act as a pro-carcinogenic factor, consistent with Dawoud et al.’s finding that SQOR is upregulated in early-stage colon cancer and highlighting the highly context-dependent nature of SQOR’s effects.

Beyond disease states, SQOR expression is also regulated by exogenous factors and contributes to cellular redox homeostasis. Zuhra et al. (2019) found that N-acetylcysteine (NAC) stimulates the H_2_S metabolic pathway in colon cancer cells (SW480) through a dual mechanism. First, NAC serves as an alternative substrate for 3-MST with a much higher catalytic efficiency than cysteine, generating N-acetylcysteine persulfide (N-AceCysSSH) and other persulfide species and thereby boosting the cells’ antioxidant capacity. Second, a 24-h NAC treatment significantly upregulated the expression and activity of both 3-MST and SQOR. This coordinated induction helps maintain redox balance in cancer cells by enhancing mitochondrial sulfide metabolism. The study systematically elucidated how NAC provides substrate and upregulates SQOR to stimulate H_2_S catabolism, highlighting SQOR’s key role in generating sulfane sulfur species and bolstering antioxidant defenses. It also suggests that NAC may act as a “double-edged sword” in cancer therapy—increasing persulfide levels to enhance antioxidant defense, but potentially supporting tumor growth in certain contexts.

Under normal intestinal physiology, SQOR is important for intestinal epithelial differentiation and homeostasis. Scheller et al. (2022) reported that during spontaneous differentiation of Caco-2 cells, SQOR mRNA and protein levels rose by 8.9-fold and 4.4-fold, respectively. This upregulation, together with increased expression of the H_2_S-producing enzyme SELENBP1 and decreased expression of CBS, forms a reciprocal regulatory network that mirrors the H_2_S detoxification mechanism of normal colonic crypt epithelium. This network enables differentiating cells to effectively handle metabolic sulfide stress from the gut microbiota. That study was the first to demonstrate SQOR’s core function in intestinal cell maturation: maintaining intestinal metabolic homeostasis by enhancing H_2_S detoxification capacity. However, in CRC cells, abnormal SQOR overexpression may become pro-carcinogenic, echoing the findings of Dawoud et al. (2024) that SQOR is upregulated early in cancer, and further emphasizing that SQOR’s role is highly situation-dependent.

SQOR also plays an important role in inflammatory bowel disease (IBD). Stummer et al. (2022) first revealed age-dependent changes in H_2_S-metabolizing enzymes in the intestinal mucosa: healthy adults had significantly lower expression of key enzymes like SQOR and 3-MST in the intestinal lining than children, suggesting that H_2_S detoxification capacity naturally declines with age. In IBD, SQOR has been identified as a key regulatory factor. In adult IBD patients, SQOR expression in the rectum and ascending colon is downregulated by ∼50% and 23%–34%, respectively, compared to healthy individuals. This decrease was not significantly correlated with inflammation severity, implying that SQOR downregulation may be an independent contributor to persistent mucosal damage in IBD. Notably, the reduction in SQOR in pediatric IBD patients (∼42% in the ascending colon) was smaller than in adults, indicating that younger patients retain some metabolic compensation—a finding that may help explain the relatively milder clinical symptoms in pediatric IBD. By simultaneously measuring five H_2_S-related enzymes (including SQOR and persulfide dioxygenase (ETHE1)), Stummer et al. demonstrated that IBD involves a systemic detoxification defect rather than merely overproduction of H_2_S, providing new insight for treatment. Building on these findings, Ma et al. (2020) showed that loss of SQOR in a mouse model of ulcerative colitis (UC) disrupts the intestinal epithelial barrier integrity and causes severe mitochondrial dysfunction. Mechanistically, SQOR preserves intestinal homeostasis through two major pathways: it inhibits Drp1-mediated mitochondrial fission, thereby reducing ROS accumulation, and it activates the *PGC1α* pathway to promote mitochondrial biogenesis and antioxidant defenses (upregulating enzymes like GPX4 and SOD2). Together, these studies demonstrate a systemic H_2_S detoxification dysfunction in IBD characterized by SQOR downregulation, which drives intestinal inflammation by upsetting mitochondrial homeostasis and exacerbating oxidative stress. Accordingly, targeting the SQOR–ROS axis is seen as a promising therapeutic strategy for UC, though the precise molecular pathways involved and the efficacy in humans remain to be further verified.

In summary, SQOR has multiple complex roles in gastrointestinal diseases. Its behavior ranges from dynamic changes in expression during CRC progression, to a detoxification function in normal intestinal epithelial differentiation, to age-dependent downregulation in IBD. Depending on the physiological or pathological context, SQOR may act as a “respiratory shield” that maintains cellular redox homeostasis and energy metabolism, or conversely, it may promote disease progression. While current studies have laid a solid foundation for understanding SQOR in gastrointestinal health and disease, there are notable limitations. Most research to date relies on *in vitro* models or public data, with a lack of *in vivo* experiments in authentic tumor microenvironments or clinical patient samples. Moreover, further work is needed to directly quantify sulfur species in tissues, decipher the specific transcriptional regulation of SQOR, and determine whether these regulatory mechanisms are universal across different diseases. Future studies integrating animal models and clinical samples are essential to explore SQOR’s dynamic regulatory network and its roles in various GI diseases—knowledge that could yield new targets and treatment strategies.

### Metabolic diseases and bone health

Emerging evidence suggests that SQOR is not confined to its traditional “detoxification” role, but is also deeply involved in skeletal metabolism and bone pathology. Maintaining SQOR homeostasis—as the rate-limiting enzyme of mitochondrial H_2_S metabolism—appears crucial for normal osteocyte function and resistance to oxidative damage. Conversely, SQOR dysfunction may be a driving factor in conditions like osteoporosis (bone density loss) and osteonecrosis (bone tissue death). Genetic studies have linked polymorphisms in the SQOR gene to susceptibility to postmenopausal osteoporosis (PMOP). For example, Cai et al. (2018) reported that the C allele of the *SQRDL* gene variant rs1044032 is associated with lower risk of PMOP (odds ratio ≈0.80), a finding validated in multiple East Asian populations. In addition, aberrant SQOR expression has been observed in bone lesions. Ma et al. (2024) found that numerous mitochondrial function-related genes, including SQOR, were significantly upregulated in patients with steroid-induced osteonecrosis of the femoral head (SONFH), implicating mechanisms such as dysregulated mitochondrial apoptosis signaling (e.g., *BCL-2*/*BAX* imbalance), increased oxidative stress, and altered energy metabolism. These studies suggest that SQOR dysfunction may directly contribute to the onset and progression of skeletal diseases. Although one study emphasized genetic susceptibility while the other focused on molecular mechanisms, both point to SQOR as a core mitochondrial H_2_S-metabolizing enzyme that is critical for maintaining bone cell function and responding to oxidative stress. Our understanding of SQOR’s role in bone metabolism is still preliminary, however. Further research is needed to determine exactly how SQOR modulates H_2_S levels to influence osteocyte survival, differentiation, and antioxidant defenses. Such work will clarify SQOR’s mechanisms in conditions like osteoporosis and osteonecrosis, and provide a foundation for early diagnosis, risk assessment, and novel targeted treatments. Illustrative features of osteoporosis and osteonecrosis related to mitochondrial redox imbalance are summarized Figure 3E.

### Reproductive system diseases

**FIGURE 4:**
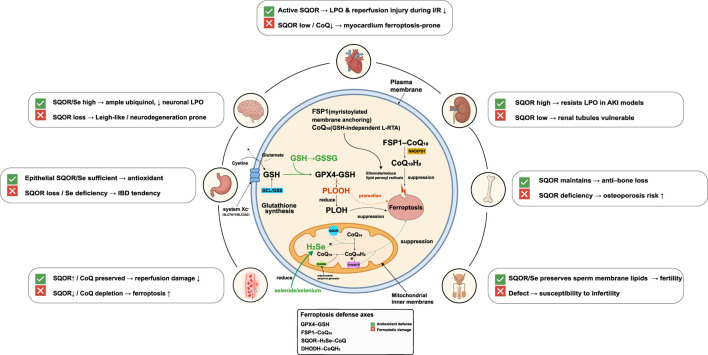
Integrated schematic of the SQOR-H_2_Se-CoQ-H_2_axis, classical ferroptosis defenses and context-dependent outcomes. Three lipid-peroxidation defense axes operate in parallel. (1) Cystine/GSH/GPX4 axis: system Xc^−^(SLC7A11/SLC3A2) imports cystine for glutathione (GSH) synthesis via glutamate-cysteine ligase (GCL) and glutathione synthetase (GSS); glutathione peroxidase 4 (GPX4) uses GSH to reduce phospholipid hydroperoxides (PLOOH) to phospholipid alcohols (PLOH), thereby suppressing ferroptosis. GSH oxidation to GSSG and GSH depletion promote ferroptosis. (2) FSP1-CoQ-NAD(P)H axis: myristoylated ferroptosis suppressor protein 1 (FSP1) at the plasma membrane reduces coenzyme Q_10_(CoQ_10_) to ubiquinol (CoQ_10_H_2_) using NAD(P)H, providing a glutathione-independent lipophilic radical-trapping antioxidant (L-RTA) that eliminates lipid peroxyl radicals. (3) Mitochondrial SQOR-H_2_Se-CoQ-H_2_axis: SQOR on the inner mitochondrial membrane uses hydrogen selenide (H_2_Se) to reduce CoQ to CoQH_2_, collaborating with dihydroorotate dehydrogenase (DHODH) and electron flow from Complex III to maintain mitochondrial CoQH_2_and suppress lipid peroxidation independently of GSH/GPX4. Organ-specific outcomes depend on SQOR/selenium/coenzyme Q availability: in the brain, high SQOR and selenium provide ample ubiquinol and low neuronal lipid peroxidation, whereas SQOR loss predisposes to Leigh-like neurodegeneration; in the heart, active SQOR reduces lipid peroxidation and reperfusion injury during ischemia-reperfusion (I/R), whereas SQOR downregulation or CoQ depletion makes myocardium prone to ferroptosis; in the kidney, high SQOR expression resists lipid peroxidation in acute kidney injury (AKI) models, whereas low expression renders renal tubules vulnerable; in the gut, epithelial SQOR/selenium sufficiency provides antioxidant protection, whereas SQOR loss or selenium deficiency leads to inflammatory bowel disease (IBD) tendencies; in bone, SQOR maintains bone mass and deficiency increases osteoporosis risk; in the male reproductive system, SQOR/selenium preserves sperm membrane lipids and fertility, whereas defects lead to infertility. SQOR, sulfide:quinone oxidoreductase; H_2_Se, hydrogen selenide; CoQ, coenzyme Q; CoQH_2_, ubiquinol; GPX4, glutathione peroxidase 4; GSH, glutathione; GSSG, oxidized glutathione; FSP1, ferroptosis suppressor protein 1; NAD(P)H, nicotinamide adenine dinucleotide (phosphate); DHODH, dihydroorotate dehydrogenase; PLOOH, phospholipid hydroperoxide; PLOH, phospholipid alcohol; LPO, lipid peroxidation; L-RTA, lipophilic radical-trapping antioxidant; I/R, ischemia-reperfusion; AKI, acute kidney injury; IBD, inflammatory bowel disease.

Long regarded as a general redox maintenance enzyme, SQOR has recently been identified as a key factor in male reproductive health. As a direct regulator of H_2_S metabolism, SQOR protein levels must be precisely kept within a narrow range to ensure normal spermatogenesis and male fertility. Any imbalance in SQOR—whether excessive accumulation or excessive degradation—will directly disrupt H_2_S homeostasis in the testes, triggering a cascade of pathological events that severely impair sperm quality and function. Elucidating how SQOR expression is regulated during spermatogenesis is therefore of great importance for understanding certain male infertility conditions. Lv et al. (2025) discovered a ubiquitin-mediated degradation pathway that governs SQOR levels to maintain H_2_S homeostasis during sperm development. Specifically, the protein ankyrin repeat and SOCS box protein 1 (ASB1) (a substrate-recognition subunit of an E3 ubiquitin ligase complex) binds to SQOR and mediates K48-linked ubiquitination of SQOR, targeting it for proteasomal degradation. This mechanism ensures that SQOR is kept at appropriate levels during spermatogenesis, preventing an excessive depletion of H_2_S. When this regulatory mechanism is impaired, SQOR accumulates abnormally, leading to a sharp decrease in testicular H_2_S and severe oxidative stress (elevated ROS levels and diminished antioxidants like glutathione and SOD). As a result, sperm DNA damage increases and morphological abnormalities rise, culminating in oligospermia, asthenospermia, and teratospermia and hence reduced fertility. Notably, supplementing exogenous H_2_S donors (such as NaHS) can effectively reverse the spermatogenic defects and infertility seen in *Asb1*-knockout mice. This finding indicates that maintaining normal H_2_S homeostasis during spermatogenesis is critical for male fertility. In fact, this study was the first to connect SQOR’s homeostatic function to the etiology of male infertility, suggesting that targeting the H_2_S metabolic pathway could be a novel therapeutic strategy for certain cases of male infertility. The ASB1-mediated K48-linked ubiquitination of SQOR and its consequences for male fertility are depicted in Figure 3F. Figure 4 presents an integrated schematic diagram of the SQOR-H_2_Se-CoQ-H_2_ axis, classical ferroptosis defense mechanisms, and context-dependent outcomes.

### SQOR targeted treatment and intervention

Research on SQOR-targeted therapies and interventions has progressed substantially in recent years. For example, in environmental remediation, genetically engineered microorganisms with enhanced SQOR activity can more efficiently degrade malodorous mercaptan pollutants (e.g., propyl mercaptan) and reduce toxic H_2_S emissions (Qiao et al., 2024). In metabolic disease contexts, studies have found that supraphysiological doses of coenzyme Q_10_ (CoQ_10_) can specifically upregulate SQOR expression and simultaneously inhibit key enzymes (CBS and CSE) in the transsulfuration pathway, thereby reshaping the serine–folate cycle and nucleotide metabolism (González-García et al., 2020). This discovery offers new insight into treating conditions such as Leigh syndrome (caused by complex I deficiency) or colorectal cancers with CBS overexpression by indirectly modulating SQOR activity via metabolic supplementation (e.g., CoQ_10_). Of course, the signaling pathways underlying this metabolic regulation (e.g., *STAT3*, HIF1α) remain to be clarified, and CoQ_10_’s low bioavailability in the brain also limits its utility in neurological diseases.

Meanwhile, a breakthrough has been made in developing direct SQOR inhibitors. Baugh et al. (2021) identified the first potent human SQOR inhibitor, with an IC_50_ of ∼29 nM. After structural optimization, this inhibitor exhibited high selectivity for SQOR over other mitochondrial enzymes, effectively avoiding off-target effects. In a mouse model of pressure-overload heart failure, the inhibitor significantly alleviated myocardial remodeling and dysfunction by inhibiting H_2_S degradation, demonstrating the feasibility of targeting the H_2_S metabolic pathway for treating heart failure. However, there is still a long journey from this “first-in-class” compound to a safe and effective drug. Its oral bioavailability and other pharmacokinetic parameters need improvement, and the potential effects of long-term SQOR inhibition on global protein persulfidation patterns have yet to be fully evaluated. Additionally, no human clinical trials of SQOR inhibitors have been initiated to date.

Nevertheless, these studies collectively highlight the vast potential of SQOR as a therapeutic target—ranging from engineered microbes for environmental bioremediation to metabolic modulation and small-molecule drugs for disease intervention—underscoring the immense promise of SQOR-targeted strategies. Future research should focus on further improving the specificity and pharmacokinetics of SQOR modulators and accelerating the translation of these strategies into clinical practice, in order to achieve breakthrough improvements in environmental health and human disease management.

## Conclusion

In summary, research on SQOR has evolved from recognizing it as an H_2_S detoxification enzyme to unveiling its complex role as a central regulator of cellular metabolism and redox homeostasis. The discovery of SQOR’s unique cysteine trisulfide bond cofactor explains its remarkable catalytic efficiency (Landry et al., 2020). SQOR dynamically regulates H_2_S levels, keeping them within an optimal physiological range: on the one hand, genetic defects (e.g., in Leigh syndrome) or CoQ deficiency that abolish SQOR activity can cause toxic H_2_S accumulation, inhibiting the mitochondrial respiratory chain and leading to severe energy failure. On the other hand, in pathological conditions such as early diabetic nephropathy, inappropriate upregulation of SQOR leads to excessive consumption of signaling H_2_S, resulting in insufficient H_2_S signaling and exacerbated tissue damage (Bushell et al., 2023). This phenomenon of “bidirectional imbalance” provides the foundation for the model of SQOR as a “metabolic rheostat.” According to this model, SQOR’s primary physiological role is not simply to eliminate H_2_S, but to maintain H_2_S concentrations within an optimal steady-state window—and when this balance is lost in either direction, opposite pathological consequences ensue.

Notably, SQOR’s functional repertoire extends beyond sulfur metabolism. SQOR can utilize the non-canonical substrate H_2_Se to reduce ubiquinone to ubiquinol, providing a GPX4-independent ferroptosis-defense pathway (Lee and Roh, 2025). This finding directly links sulfur metabolism, selenium metabolism, and programmed cell death, and it also helps explain SQOR’s dual roles in different cancers. In hypoxic tumors such as pancreatic cancer, high SQOR expression enhances resistance to ferroptosis, helping tumor cells tolerate oxidative stress (a pro-tumorigenic effect). In contrast, in immunologically active tumors like osteosarcoma, high SQOR expression boosts anti-tumor immune activity and inhibits tumor progression (an anti-tumorigenic effect) (Wang Y. et al., 2024). This stark contrast depends on the tumor’s metabolic demands and immune microenvironment, indicating that SQOR’s role is highly context-dependent. Consequently, a “one-size-fits-all” SQOR-targeted therapy is not feasible. Before clinical application, it will be critical to develop reliable companion diagnostics to determine the activity state of SQOR in a patient’s lesion and the cell’s dependence on SQOR, in order to guide whether SQOR should be inhibited or activated for therapy.

The successful development of the first highly selective SQOR inhibitors demonstrates that SQOR is indeed a “druggable” target, opening a new paradigm for treating diseases like heart failure (Jackson et al., 2022). However, a significant gap remains between these early chemical probes and a safe, effective drug—current inhibitors’ pharmacokinetics and long-term safety require further optimization and evaluation, and no clinical trial data on SQOR-targeted therapy are yet available. More importantly, as illustrated by the “selenium–anticancer paradox,” protective effects observed in preclinical models do not guarantee similar outcomes in the complex human organism. This highlights the absolute necessity of developing robust *in vivo* monitoring methods for H_2_S and its metabolites to stratify patients and monitor treatment responses before advancing SQOR-targeted therapies.

However, despite these encouraging advances, several limitations hamper the clinical translation of SQOR modulators. First, most available compounds exhibit suboptimal pharmacokinetic profiles and poor bioavailability, reflecting the difficulty of delivering drugs to the mitochondrial matrix where SQOR resides. Second, although selectivity over other mitochondrial enzymes has improved, off-target interactions with related sulfide oxidation pathways or broader redox networks remain a concern and need comprehensive screening. Finally, sustained suppression or activation of SQOR could disrupt H_2_S homeostasis and mitochondrial redox balance; thus the long-term safety and potential systemic toxicity of SQOR modulation must be rigorously evaluated in chronic preclinical models before progressing to human trials.

Looking ahead, SQOR research is at a critical turning point. To resolve core controversies such as “What is SQOR’s primary sulfur acceptor *in vivo*?” and “What molecular switches dictate SQOR’s different roles in different diseases?”, we must shift from reductionist approaches to systems-level investigations. This will involve multi-omics integration and advanced metabolic imaging to comprehensively analyze the sulfur/selenium metabolism, energy metabolism, and redox networks in which SQOR is embedded. For example, targeted metabolomics to directly quantify sulfur metabolites in the mitochondrial matrix of different cell types could provide definitive answers to the sulfur acceptor question. Integrating transcriptomic, proteomic, metabolomic, and clinical data to construct network models will reveal how SQOR interacts with other pathways and influences disease progression across pathological states. Such efforts will require close collaboration among molecular biologists, biochemists, bioinformaticians, computational biologists, and clinicians. By collectively analyzing multidimensional data and dynamically simulating SQOR’s regulatory network, we can ultimately elucidate SQOR’s complex roles in health and disease and translate this knowledge into new strategies to benefit both the environment and human health. For instance, specific SQOR modulators could be developed and used to precisely restore H_2_S homeostasis in patients, leading to breakthrough therapies for various diseases.

In conclusion, SQOR is a key enzyme in sulfur homeostasis that is gradually revealing enormous potential as a therapeutic target for disease diagnosis and treatment. This warrants further exploration of SQOR and proactive efforts to expand its applications in biomedical research and beyond.

## Glossary

ALS: Amyotrophic lateral sclerosis
ASB1: Ankyrin repeat and SOCS box protein 1
ATP: Adenosine triphosphate
BAX: BCL-2-associated X protein
BCL-2: B-cell lymphoma 2
CBS: Cystathionine β-synthase
**CoQ/CoQ**_**10**_: Coenzyme Q/Coenzyme Q10
**CoQ**_**10**_ **H**_**2**_: Ubiquinol (reduced CoQ_10_)
CRC: Colorectal cancer
cGAS: Cyclic GMP–AMP synthase
cGAS–STING: cGAS–stimulator of interferon genes pathway
CSE: Cystathionine γ-lyase
DHODH: Dihydroorotate dehydrogenase
DNA: Deoxyribonucleic acid
Drp1: Dynamin-related protein 1
ELOB: Elongin B
ETC: Electron transport chain
ETHE1: Persulfide dioxygenase (ethylmalonic encephalopathy protein 1)
FAD: Flavin adenine dinucleotide
FSP1: Ferroptosis suppressor protein 1
GPX4: Glutathione peroxidase 4
GSH: Glutathione
GSSH: Glutathione persulfide
GSSSG: Glutathione trisulfide
**H**_**2**_ **S**: Hydrogen sulfide
**H**_**2**_ **Se**: Hydrogen selenide
HIF-1α: Hypoxia-inducible factor-1 alpha
**IC**_**50**_: Half-maximal inhibitory concentration
IBD: Inflammatory bowel disease
IFN: Interferon
MPST/3-MST: 3-Mercaptopyruvate sulfurtransferase
NaHS: Sodium hydrosulfide
NLRP3: NLR family pyrin domain containing 3
NQO1: NAD(P)H:quinone oxidoreductase 1
NRF2: Nuclear factor erythroid 2–related factor 2
PC: Pyruvate carboxylase
PGC1α: Peroxisome proliferator-activated receptor-γ coactivator-1α
Qred/Qox: Reduced/oxidized quinone
ROS: Reactive oxygen species
SOD/SOD2: Superoxide dismutase/Superoxide dismutase 2
SONFH: Steroid-induced osteonecrosis of the femoral head
SQOR: Sulfide:quinone oxidoreductase
STAT3: Signal transducer and activator of transcription 3
STING: Stimulator of interferon genes
SUOX: Sulfite oxidase
TST: Thiosulfate sulfurtransferase
UC: Ulcerative colitis
xCT (SLC7A11): Cystine/glutamate antiporter
